# Fracture Models and Effect of Fibers on Fracture Properties of Cementitious Composites—A Review

**DOI:** 10.3390/ma13235495

**Published:** 2020-12-02

**Authors:** Peng Zhang, Yonghui Yang, Juan Wang, Meiju Jiao, Yifeng Ling

**Affiliations:** 1 School of Water Conservancy Engineering, Zhengzhou University, Zhengzhou 450001, China; zhangpeng@zzu.edu.cn (P.Z.); yang1287820@163.com (Y.Y.); jiaomj@zzu.edu.cn (M.J.); 2 Department of Civil, Construction and Environmental Engineering, Iowa State University, Ames, IA 50011, USA; yling@iastate.edu

**Keywords:** cementitious composites, model, polyvinyl alcohol fiber, steel fiber, fracture property

## Abstract

Cementitious composites have good ductility and pseudo-crack control. However, in practical applications of these composites, the external load and environmental erosion eventually form a large crack in the matrix, resulting in matrix fracture. The fracture of cementitious composite materials causes not only structural insufficiency, but also economic losses associated with the maintenance and reinforcement of cementitious composite components. Therefore, it is necessary to study the fracture properties of cementitious composites for preventing the fracture of the matrix. In this paper, a multi-crack cracking model, fictitious crack model, crack band model, pseudo-strain hardening model, and double-K fracture model for cementitious composites are presented, and their advantages and disadvantages are analyzed. The multi-crack cracking model can determine the optimal mixing amount of fibers in the matrix. The fictitious crack model and crack band model are stress softening models describing the cohesion in the fracture process area. The pseudo-strain hardening model is mainly applied to ductile materials. The double-K fracture model mainly describes the fracture process of concrete. Additionally, the effects of polyvinyl alcohol (PVA) fibers and steel fibers (SFs) on the fracture properties of the matrix are analyzed. The fracture properties of cementitious composite can be greatly improved by adding 1.5–2% PVA fiber or 4% steel fiber (SF). The fracture property of cementitious composite can also be improved by adding 1.5% steel fiber and 1% PVA fiber. However, there are many problems to be solved for the application of cementitious composites in actual engineering. Therefore, further research is needed to solve the fracture problems frequently encountered in engineering.

## 1. Introduction

Cementitious composites were proposed in the 1990s by Victor Li et al. [1,2,3], who used micromechanics and fracture mechanics to study the properties of the matrix, the properties of the fiber, the properties of the interface between the fiber and the matrix, and their interrelationships. The crack stress criterion and crack steady-state expansion criterion were established. A pseudo-strain-hardening tensile phenomenon occurred when the staple fiber was added to the matrix, which improved the stress–strain characteristics of the matrix. When cementitious composites are subjected to external loads, the internal or newly formed micro-cracks in the matrix gradually expand until the specimen is destroyed [4].

The toughness of concrete can be improved by adding PVA fiber, steel fiber, polypropylene fiber, polyethylene fiber, and natural fiber (flax/wool twine) into concrete. Ghaffar et al. [5] added 1% flax/wool twine to the cementitious material and compared and analyzed the influence of epoxy (EP) or polyurethane (PU) coated on the surface of flax/wool twine on the compressive strength and flexural strength of the matrix. It was found that flax/wool twine coated with EP or PU could significantly improve the mechanical properties of the matrix. This may be a natural fiber surface coating that makes the fiber bond more tightly to the matrix. Polypropylene fiber has the advantages of low density, high wet strength, high elongation, good alkali resistance, and low price. The addition of polypropylene fiber into concrete can reduce the early shrinkage deformation of concrete, prevent the plastic shrinkage cracking, and improve the impermeability of concrete. Adding the proper amount of polypropylene fiber into concrete can improve the fracture performance, strength, and toughness of concrete. Therefore, polypropylene fiber has been widely used in cementitious composites. The content of polyvinyl alcohol fibers in cementitious composites generally does not exceed 2%. The addition of steel fibers, PVA fibers, or polyethylene (PE) fibers to traditional cementitious composites can increase the strain capacity of the substrate by 3–5% [6,7,8]. This is 300–500 times the strength of ordinary concrete, but the compressive strengths of cementitious composites are lower than that of ordinary concrete.

In the process of strain hardening, cementitious composites exhibit multiple cracks and absorb more fracture energy and have a better impact resistance than ordinary concrete. Cementitious composites can control their own crack width; the average width is ≤100 μm, and the crack spacing is 3–10 mm [9,10]. If the cracks in the PVA-cementitious composite are no wider than 50 μm, all the cracks can heal. If the crack width is in the range of 50–150 μm, only part of the crack can heal. A crack having a width of >150 μm is difficult to heal [11]. Owing to the high-strength, high-elastic modulus and nontoxic hydrophilic PVA fibers the brittleness of the cementitious composite is significantly improved, while the fracture energy of the material is increased, which is useful for solving the durability problem caused by the high brittleness of cementitious composites. These features of cementitious composites can also reduce the concrete brittleness and ease of fracture and mitigate other shortcomings.

Cementitious composites contain numerous cementitious materials and do not contain coarse aggregates, making their elastic moduli lower than that of ordinary concrete [12]. Cementitious composites have tensile strains of 6–10%, tensile strengths of 6–16 MPa, and compressive strengths of 43–115 MPa [6,13,14]. Because of these properties, cementitious composites are widely used. The fatigue performance and deformation capacity of cementitious composites are superior to those of ordinary concrete and fiber-reinforced concrete. Cementitious composites have better applicability application value than ordinary concrete with regard to some aspects, e.g., impact resistance for seismic structures, dams, and irrigation channels with numerous cracks and durability for road maintenance and bridge foundation [15,16]. In 2002, engineers used cementitious composites to repair the panels of a highway bridge in Michigan, USA. After two years of use and exposure to the harsh winter environment, the crack width of the cementitious composite bridge deck was controlled below 30 μm and had a good working condition [17]. Therefore, cementitious composites are useful for practical applications.

When the engineering structure is subjected to a large external force, the internal micro-cracks in the structure gradually expand into large cracks until the cracks run through the entire structure and the specimens rupture, which not only affects the safety of the building structure, but also causes economic losses. Thus, fracture has always been a popular topic in civil engineering. In the 1950s, researchers developed fracture mechanics theory according to analysis of brittle fracture accidents in engineering structures under low stress and components containing macroscopic cracks. In 1961, Kaplan applied fracture mechanics theory to concrete for the first time, and determined the fracture toughness parameters of concrete using the fracture mechanics method [18].

Though the multi-crack cracking (ACK) model, fictitious crack (FC) model, pseudo-strain (PSH) model, double-K fracture (DKF) model, and two-parameter fracture model have been widely used, they have certain drawbacks. For example, in the process of multi-fracture analysis of fiber-reinforced cementitious composites, the existence of frictional shear stress transfer and elastic stress transfer between fiber-reinforced cementitious composites are not considered in ACK mode. The stress softening model of FPZ (fracture process zone) is used to describe the fracture expansion in both FC model and CB model used; however, the corresponding analytical solutions are lacking. Additionally, the calculation process of the PSH model is too complicated. Furthermore, the linear propagation of fractures is not considered in the DKF model. The two-parameter fracture (TPF) model measured whether concrete cracked or not using the stress intensity factor and fracture tip opening displacement. The advantage of the TPF model is that it can obtain the analytic solution of the two-parameter critical fracture criterion, but the disadvantage does not take into account the influence of FPZ on the final critical instability during the fracture process. Although these models have some shortcomings, they are still of great value in practical engineering applications.

The crack propagation degree of concrete is determined by the fracture toughness, which reflects the crack resistance of specimens under an applied load [19,20]. Under external loads, cementitious composites exhibit multiple cracks; additionally, they can absorb more energy and have greater ductility than ordinary concrete. Herein, the fracture properties of cementitious composites are reviewed. Cementitious composite fracture models, such as the ACK model, FC model, CB model, PSH model, and DKF model, are introduced and analyzed. The purpose of analyzing the advantages and disadvantages of these models is to solve and improve the structural fracture problems in practical engineering by selecting appropriate models to analyze these problems. In the mix and structural design of cementitious composites, according to these fracture models, the appropriate amount of fibers, aggregate, and other materials can be selected, as well as the appropriate size of the specimen, so as to improve the fracture performance of the structure. Furthermore, the effects of PVA fibers and SFs on the fracture performance of cementitious composites are investigated.

## 2. Fracture Models

### 2.1. ACK Model

Brittle cracking is an important factor affecting the durability of concrete. Multi-crack cracking is one of the most effective measures to improve the toughness, limit tensile strain, and reduce brittleness of fiber-reinforced cementitious composites. Therefore, it is necessary to study the multi-crack cracking model of fiber-reinforced cementitious composites. Cementitious composites exhibit the following multi-crack behavior, under the action of an axial tensile load; as the load increases, cracks begin to appear in the matrix. However, the fibers can transfer the stress at the crack to the un-cracked matrix and provide bridging stress until a new crack (a steady-state crack) is generated in the matrix. In this process, until the crack penetrates the entire section, the stress and deformation fields at the crack tip do not change, and multiple cracks can improve the toughness and ultimate tensile strain of cementitious composites [21]. To better study the fracture performance of cementitious composites, Aveston et al. proposed a multi-crack cracking model of fiber-reinforced cementitious composites called the ACK model (i.e., bending load-deflection curve model of a thin plate) [22].

The ACK model can be described as follows:

(1) $$V_{f}>\frac{E_{c}\varepsilon_{m}^{u}}{\sigma_{f}^{u}}$$

(2) $$\sigma_{f}^{u}V_{f}>\sigma_{m}^{u}V_{m}+\sigma_{f}^{\prime }V_{f}$$

where $V_{f}$ and $V_{m}$ represent the volume ratios of the fibers and cementitious composites, respectively; $E_{c}$ represents the elastic modulus of the cementitious composites; $E_{m}^{u}$ represents the ultimate tensile strain of the cementitious composites; $\sigma_{f}^{u}$ represents the tensile strength of the fiber; $\sigma_{m}^{u}$ represents the tensile strength of the cementitious composites; and $\sigma_{f}^{\prime }$ represents the tensile stress of the fiber when the cementitious composites reach $\sigma_{m}^{u}$.

Equations (1) and (2) must satisfy the following conditions. The tensile strength and ultimate elongation of the continuous fibers must be sufficient, and the distribution direction of the fiber in the brittle cementitious composites must be one-dimensional. If the volume rate of the fiber is greater than its critical volume rate, and the tensile stress of the fiber is higher than the tensile strength of the matrix, the matrix exhibits a multi-crack phenomenon. Using Equation (1), the ultimate tensile strain of the cementitious composite ($\varepsilon_{m}^{u}$) is determined as follows:

(3) $$\varepsilon_{m}^{u}={\left[\frac{12\tau \gamma_{m}E_{f}V_{f}^{2}}{E_{fc}E_{m}^{2}\gamma_{f}V_{m}}\right]}^{1/3}$$

Here, the coefficient $\gamma_{m}$ can be expressed as

(4) $$\gamma_{m}=0.5\frac{K_{Ic}^{2}}{E_{m}}$$

where τ represents the average shear strength of the fiber and cement matrix; $E_{f}$ and $E_{m}$ represent the moduli of the fiber and cementitious composite, respectively; $\gamma_{f}$ represents the fiber radius; and $K_{Ic}$ represents the critical stress intensity factor of the fracture tip. The presence of fibers can result in cracks in the cementitious composite, increasing the strain $\varepsilon_{m}^{u}$. $\varepsilon_{m}^{u}$ exhibits a positive correlation with $V_{f}$ and τ and a negative correlation with $\gamma_{f}$.

When a crack occurs in a cementitious composite, the entire load on the cracking surface of the matrix is transferred to the fiber across the crack. The fiber transfers the load to the un-cracked matrix on both sides of the crack through the shear bond of the matrix interface until the tensile strength of the cracked matrix is reached, and additional cracks are generated in the matrix. The reciprocating stress transfer between the fiber and the cementitious composite eventually results in multiple cracks in the cementitious composite, with minimum and maximum spacings of $X^{\prime }$ and 2$X^{\prime }$, respectively. $X^{\prime }$ can be expressed as follows:

(5) $$X^{\prime }=\frac{V_{m}d_{f}\sigma_{m}^{u}}{4V_{f}\tau }$$

where *d_f_* represents the fiber diameter.

After multiple cracks are formed in the cementitious composite, the upper and lower limits of the tensile strain can be expressed as follows:

(6) $$\varepsilon_{m}^{u}(1+\alpha /2)<\varepsilon_{c}^{mc}<\varepsilon_{m}^{u}(1+3\alpha /4)$$

where $\varepsilon_{c}^{mc}$ represents the strain of the one-dimensional continuous fiber-reinforced cementitious composite at the end of multi-crack cracking. The coefficient α is calculated as follows:

(7) $$\alpha =E_{m}V_{m}/E_{f}V_{f}$$

**Figure 1:**
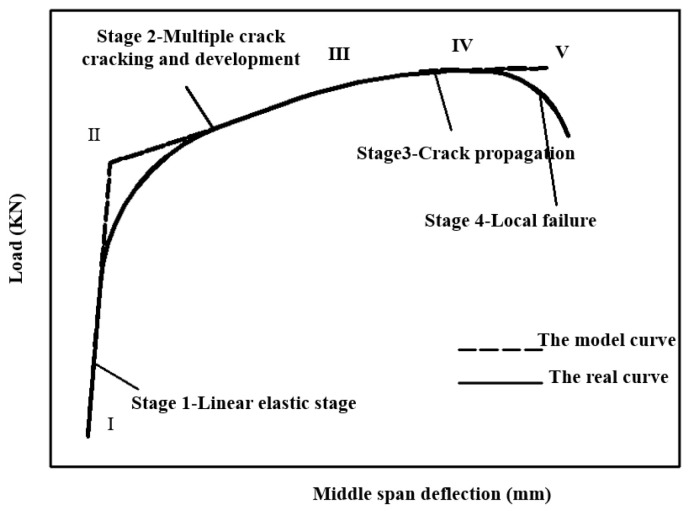
Bending load–deflection curve model of thin plate (ACK model).

It can be seen from Figure 1, according to the changes of fiber toughening effect and crack development mode, that the specimen can be roughly divided into four stages from the initial stress to the final failure. Stage 1 is the elastic stage, in which the external force borne by the matrix is much larger than that of PVA fiber, and the deformation of the material is composite Hooke’s law. The end point of this stage is that the first crack appears in the matrix, that is, the stress of the composite material reaches the cracking strength point (bending proportional strength limit point). Stage 2 is the multiple-crack cracking and development stage (also known as the yield stage). At this stage, stress is transferred back and forth between the matrix and the fiber, resulting in a large number of fine cracks in the matrix. When the specimen no longer produces new cracks, this stage ends, and the end point is also called the yield point. Yield points are sometimes determined by the Feng et al. method [23]. Stage 3 is the crack propagation stage, in which no new cracks are generated. However, the crack width and mid-span deflection of the specimen continue to increase until the weakest crack of the specimen is rapidly destroyed. If this stage is longer, it indicates that PVA fiber has a good effect on the matrix, the crack width in the specimen is small, and the deflection of the specimen is also large, with a good deformation effect. The increase of fly ash content will also increase the peak load and deformation capacity of specimens [24]. Stage 4 is the local failure stage. At this stage, the bearing capacity of the specimen decreases, most of the fibers on the fracture surface of the specimen will be pulled out, and the specimen will be destroyed. Although the ACK model curve is somewhat different from the actual curve, it can well reflect the mechanical state of the matrix and provide guidance for the design of thin plate’s load and deflection.

According to the foregoing analysis, the ACK model is based on a one-dimensional directional continuous fiber-reinforced cementitious composite, and the optimum fiber content can be calculated. The ACK model has the following disadvantages. It does not consider the existence of frictional shear stress or the elastic stress transfer between the fiber and the cementitious composite. Additionally, the effects of the fiber orientation and length on the stress–strain curve are not considered, and the failure mode of the fiber (e.g., pullout or breakage) is not considered. The calculation accuracy of load and deflection in the ACK model also needs to be improved, otherwise it will affect the application of practical engineering. Although the ACK model has shortcomings, it plays a significant role in the improvement of the multi-fracture model and the development of high-performance fiber-reinforced cementitious composites.

### 2.2. FC Model

Before the initial unstable propagation of the concrete crack, the development of the fracture zone at the leading edge of the crack end leads to the stable propagation of the crack, which is called subcritical propagation. The crack end of the initial crack expands subcritically; thus, the critical stress concentration and strength factor at the crack end are reduced [25]. The cohesive crack method and the equivalent elastic crack method are the two main methods for describing the crack growth behavior and stress distribution near the crack tip [26]. Between them, the cohesive crack method is more widely used. The FC model of concrete is a typical cohesive crack model.

**Figure 2:**
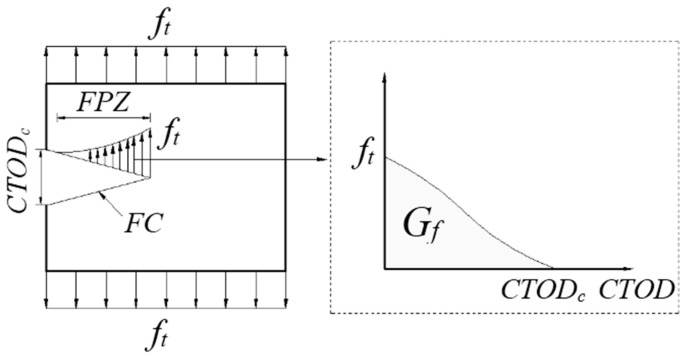
FC model.

**Figure 3:**
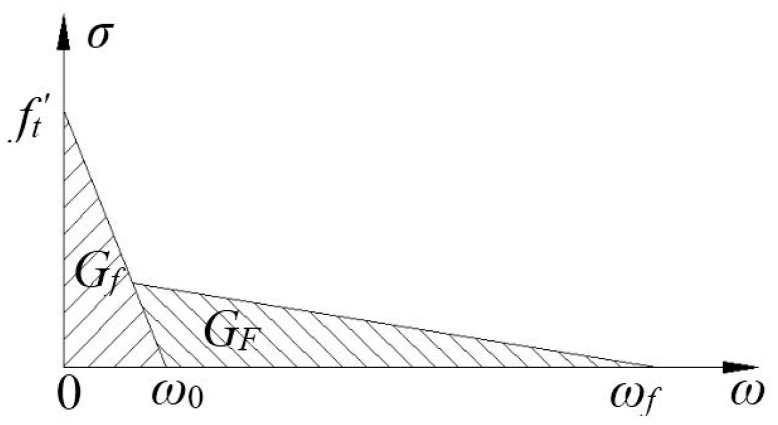
Bilinear tensile softening curve of concrete.

The FC model was proposed by Hillerborg et al. [27]. Its characteristic is that the stress at the crack tip of concrete is non-singular, and there are many micro-crack areas, which is called the fracture process zone (FPZ). The FPZ in concrete can be replaced by a fictitious crack. When the stress at the fictitious crack tip reaches the tensile strength ($f_{t}$) of the matrix, the fictitious crack begins to expand. FPZ and $f_{t}$ are perpendicular to each other. There exists cohesive stress on the fictitious crack surface, and the cohesive stress decreases with the increase of crack width. The cohesive stress and crack opening follow the softening curve of concrete, as shown in Figure 2 and Figure 3. According to elastic modulus (E), $f_{t}$ and fracture energy ($G_{f}$), the tensile softening curve of concrete can be calculated, and then the FC model can be established [28]. Figure 1 shows the feature curve of the FC model, when the crack is in a critical state, the fictitious crack tip stress reaches the tensile strength of the concrete material $f_{t}$, the crack opening displacement is maximized, and the stress is 0. Research results for the FC model indicated that the subcritical expansion of concrete cracks before their failure is the main reason for the size effect of the concrete fracture toughness [29].

As shown in Figure 2, it is assumed that only the fracture area of concrete consumes energy. The size-independent fracture energy $G_{f}$ can be expressed as follows:

(8) $$G_{f}=\frac{1}{2}f_{t}\left(\frac{CTOD_{c}}{l_{r}}\right)l_{r}=\frac{1}{2}f_{t}(CTOD_{c})$$

where $CTOD_{f}$ represents the critical crack tip opening displacement, $f_{t}$ represents the tensile strength, and $l_{r}$ represents the height of the plate.

**Figure 4:**
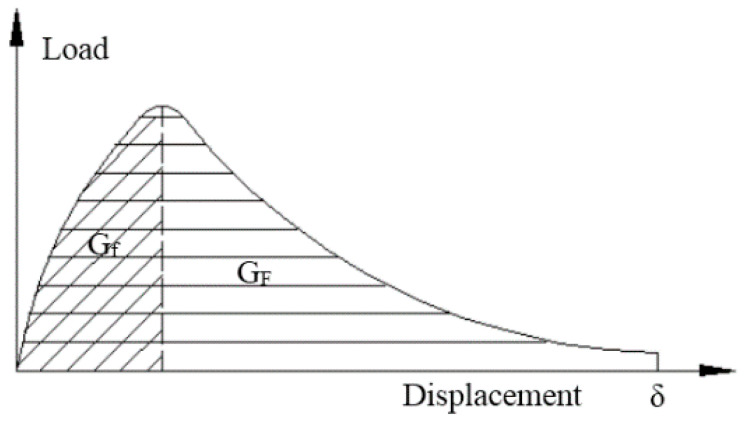
Difference between $G_{f}$ and $G_{F}$ in the load–displacement curve [30].

According to the bilinear softening curve shown in Figure 3, the FC model has two types of fracture energies: $G_{F}$ and $G_{f}$. $G_{F}$ and $G_{f}$ can be expressed as follows:

(9) $$G_{F}=\int_{0}^{\infty }f(w)dw$$

(10) $$G_{f}=\frac{f_{t}^{\prime }{}^{2}}{2\sigma_{0}^{\prime }}=\frac{w_{0}^{2}\sigma_{0}^{\prime }}{2}$$

(11) $$\sigma_{0}^{\prime }=\frac{df(0)}{dw}$$

where the size-dependent fracture energy ($G_{F}$) is given by the area under the curve f(w); $G_{F}$ is given by the area of the curve from the beginning of its slope to $\sigma_{0}^{\prime }$, within the actual size of the structure; $f_{t}^{\prime }$ represents the direct tensile strength of the concrete; $G_{f}$ affects the maximum load of the structure; w represents the crack opening displacement; and $w_{0}$ represents the crack opening displacement when FPZ cohesion drops to 0. $G_{F}$ is equal to the total fracture energy in the softening curve of ordinary concrete. Under the effects of different sizes, the energy consumption for different specimen masses and the energy consumption of the FPZ cracks, $G_{F}$ changes. The difference between $G_{F}$ and $G_{f}$ is shown in Figure 4. It is necessary to determine the $G_{F}/G_{f}$ and $G_{F}/G_{f}$ ratios for ordinary concrete, which should be in the range of 1.5–3.5 [30].

The FC model is based on the stress-deformation characteristics of the concrete under tension. The tensile softening curves of the FC model are usually used as nonlinear and bilinear curve, and the nonlinear softening relationship is generally used when numerical simulations and an infinite plate central crack model are adopted. The FC model is relatively simple and easy to understand. It can simulate the complex nonlinear phenomena of concrete FPZ, and can predict the local real physical phenomena near the crack and the crack tip. The FC model is mainly used for open-type cracks (I) but has recently been used for slip-open crack (II) and split crack (III) models.

Choubey et al., using FC model, studied the effect of recycled aggregate (RA) content of 30%, 50%, 70%, and 100% on the fracture energy and E of the matrix. It was found that the fracture energy of recycled aggregate concrete decreased by 4.5%, 8.3%, 12.0%, and 17.6%, respectively, compared with that of ordinary concrete [29]. The FC model can be used to predict the bending moment bearing capacity of fiber reinforced concrete beams well and easily obtain the ultimate load under beam bending failure [31]. By using the FC model and uniaxial tensile test, Fantilli et al. measured the critical value of the content of PVA fiber and PE fiber as 0.75%, which can improve the stress–strain performance and splitting tensile strength of cementitious composites and meanwhile reduce the cost of the matrix [32]. Therefore, the FC model is a practical model.

However, the FC model also has some disadvantages. The governing equation for solving the FC model’s fracture process area is a nonlinear singular integral equation, which makes it difficult to calculate the distribution expressions of its displacement and cohesive force [33]. The FC model adopts the subcritical growth length as the effective crack growth length, but ignores the initial damage of concrete in the process of pouring and forming, which will also affect the effective crack growth length of concrete. The analytical solution of the propagation length of the fracture zone cannot be obtained using the FC model. The fracture length in the FC model is directly reduced to a straight line, which is not common in practice. In the case of uniaxial stretching, the accuracy and rationality of concrete softening curve measurement have some errors.

### 2.3. CB Model

The CB model, which is also known as the blunt fracture zone model, was proposed by Bažant. The CB model assumes that the FPZ of the main crack and its tip is a micro crack cross-section with a certain width and continuous distribution. Fracture propagation is composed of strain softening and expansion of the micro-crack zone. The concepts of the stress softening curve and fracture energy of FC model are introduced. When the tensile stress of the concrete in the crack zone reaches its tensile strength, it will enter the softening stage, while the material outside the crack zone still maintains the linear elastic property. The energy consumed by the complete cracking of the crack zone is the fracture energy of concrete. The mechanical properties used in this model are consistent with the continuous damage mechanics, and the crack stability analysis problem is transformed into the deformation problem for continuous media [34]. The specimens in the CB model and the loads acting on the specimens are symmetric.

**Figure 5:**
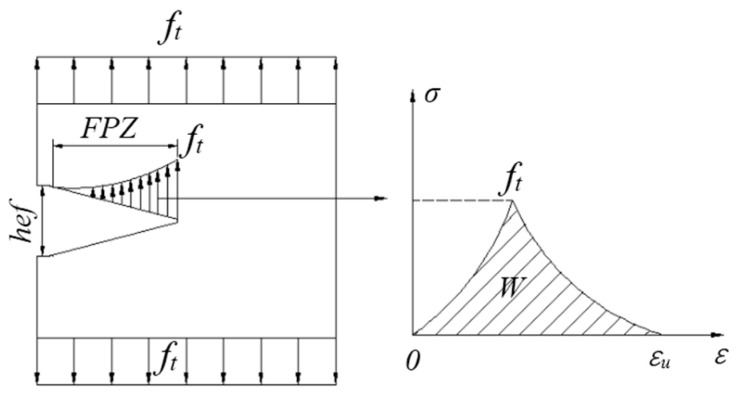
CB model.

As shown in Figure 5, the CB model is mainly related to $G_{f}$ (fracture energy), $f_{t}$ (tensile strength), and $h_{ef}$ (crack band width). The optimal value of $h_{ef}$ is 2–3 times the biggest aggregate particle size in the concrete [27], there is a certain empiricism in this. For a concrete slab with a gap or crack, if the energy consumption in the unit of the slab is correctly calculated, the gap or crack has little influence on the calculation results of the CB model. The unit energy loss for the crack zone is independent of the unit size of the CB model, mainly because the ratio $h_{ef}/l_{ch}$ (characteristic length in the FPZ) affects the slope of the post-peak softening curve. In the finite-element calculation process, the ratio $h_{ef}/l_{ch}$ is used to scale the post-peak part of the main composition to the appropriate level, and the strain of the fracture curve becomes steep; however, the unit energy loss remains constant [35]. The calculation formula for $G_{f}$ in the CB model is

(12) $$G_{f}=wh_{ef}$$

where w represents the strain energy density, and $h_{ef}$ represents the crack band width or finite-element size.

The CB model can automatically form new cracks, which do not need to be re-divided into cells. Fracture direction is not affected by cell division direction. Cell size has no effect on CB model analysis results. CB model has high accuracy in calculation. The CB model regards the fracture zone as an orthotropic medium, which can easily determine the stress and deformation of the fracture zone and structure.

The core theories of the CB and FC models are based on the tensile softening curve of the concrete, the fracture energy, tensile strength $f_{t}$, and FPZ. Both the FC and CB models are nonlinear fracture mechanics models of concrete; i.e., they do not follow linear elastic fracture mechanics (LEFM). The numerical methods usually used in the two models are both finite element methods, but they lack the corresponding analytical solutions. However, the crack initiation criterion of the CB and FC models are that the stress reaches the tensile strength ($f_{t}$) of the matrix, respectively. For the mechanical treatment of the micro-crack area, the FC model is mainly characterized by a bilinear strain softening curve, the CB model is characterized by a uniform and parallel distribution of micro-crack, and the damage degree is reflected by the reduction in the elastic modulus. These models do not solve the problem of numerical instability caused by the superposition of positive and negative stiffnesses in the soft and hard regions; thus, further research is necessary. Thus, the FC and CB models have similarities and differences.

### 2.4. PSH Model

Cementitious composites under direct tensile loads produce multiple fine cracks, which is also a characteristic of the PSH of cementitious composites. The strain hardening behavior of cementitious composites should satisfy both the strength criterion and the energy criterion; otherwise, multiple cracks do not occur in the cementitious composite, and it directly enters the softening stage. The softening phenomenon also occurs in ordinary fiber-reinforced concrete [36]. The strength criterion of cementitious composites can be expressed as follows:

(13) $$\sigma_{c}=\sigma_{0}$$

where $\sigma_{0}$ represents the maximum bridge bonding force of the fibers. Owing to the uneven distribution of the fibers in the matrix, the values of $\sigma_{0}$ on different crack surfaces are different. $\sigma_{c}$ represents the initial tensile cracking stress of the matrix.

**Figure 6:**
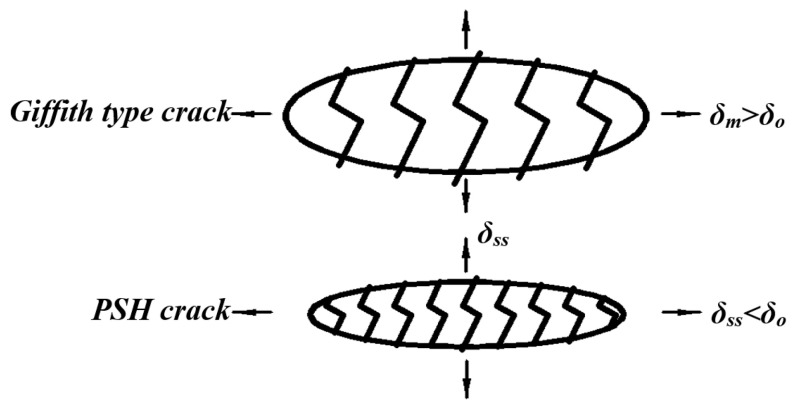
Crack propagation characteristics.

If the conditions in cementitious composites satisfy Equation (13), multiple cracks occur in the matrix. Failure to satisfy Equation (13) results in localized fracture failure of the Griffith type, which is common in fiber-reinforced concrete, as shown in Figure 6. Equation (13) requires that the initial tensile cracking stress of the matrix be less than the maximum bridge connection force of the fiber; otherwise, the fiber is broken or pulled out. If the applied load cannot be transferred to the cementitious composite through the fiber, no new cracks can be generated in the matrix. The strength criterion does not apply to the case where the initial defect size of the specimen is too small or the fracture toughness of the matrix is too high.

**Figure 7:**
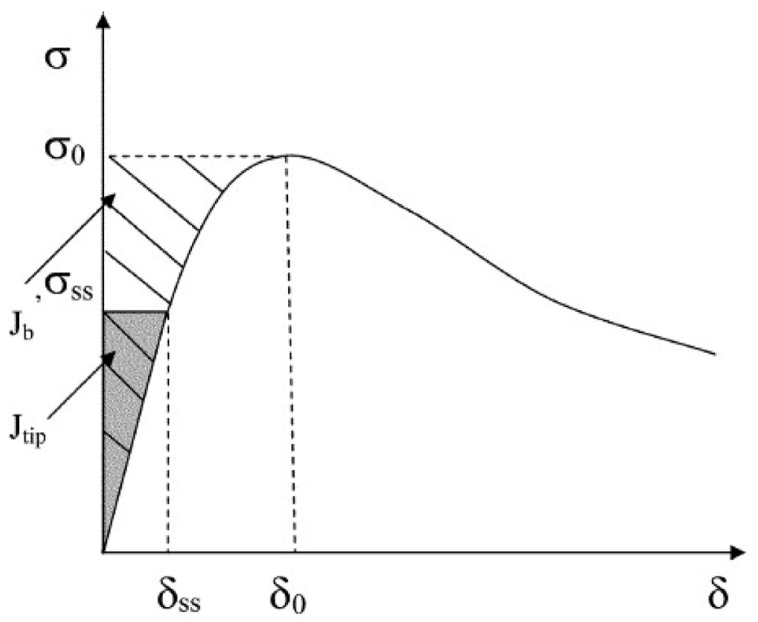
Typical *σ*-*δ* constitutive relation for the cementitious composite [38].

The number of bridging fibers on the crack surface in the matrix is ≥1000 (per square centimeter); thus, the bridging fibers can bear a large external load. The crack direction in the cementitious composite is random, while the fibers bridging cracks teds to adjust their direction according to the tensile stress, and the bridging fibers usually bend close to the matrix. Bridging fibers usually exhibit partial shedding and extension. With an increase in the external load, most of the fibers are pulled out or broken [37]. The bridging function of the fibers significantly affects both the strength and energy criteria. The relationship between the bridging force of the bridging fiber and the crack opening displacement is shown in Figure 7. The repeated formation of steady-state cracks leads to the formation of multiple cracks and PSH in cementitious composites [38]. Marshall and Cox [39] analyzed the steady-state cracking conditions of the matrix by using the J-integral method. The crack width did not increase with an increase in the distance from the crack tip, except in the vicinity of the crack tip. The steady-state cracking condition of the matrix is also known as the energy criterion of PSH and can be expressed as follows:

(14) $$\sigma_{ss}\delta_{ss}-\int_{0}^{\delta_{ss}}\sigma (\delta )d\delta =J_{tip}$$

(15) $$\sigma_{0}\delta_{0}-\int_{0}^{\delta_{0}}\sigma (\delta )d\delta =J_{b}^{\prime }\geq J_{tip}$$

(16) $$J_{tip}\approx K_{m}^{2}/E_{m}$$

where $J_{b}^{\prime }$ represents the complementary energy which is the net energy required for fracture propagation; $\sigma_{0}$ represents the crack opening corresponding to the maximum bridge connection force $\delta_{0}$; $J_{tip}$ represents the fracture energy at the crack tip; $\delta_{ss}$ represents the steady-state crack opening stress corresponding to the steady-state crack opening displacement $\sigma_{ss}$; $K_{m}$ represents the fracture toughness of the matrix; and $E_{m}$ represents the elastic modulus of the matrix. $K_{m}$ is affected by the type and size of the aggregate in the matrix material, the water–cement ratio (w/c), the type of binder, and other factors. A larger aggregate particle size corresponds to more tortuous crack paths in the matrix and larger values of $K_{m}$ and $J_{tip}$. $J_{b}^{\prime }$ is mainly affected by the chemical and friction bonds in the fiber/matrix interface [40].

The multi-crack behavior and ductility of the cementitious composite are related to the PSHE ($J_{b}^{\prime }/J_{tip}$) and PSHs ($\sigma_{0}/\sigma_{c}$) of the matrix. $J_{b}^{\prime }\geq J_{tip}$ can make this crack growth mode better than Giffith’s crack growth mode. Increasing $J_{b}^{\prime }$ contributes to the formation of multiple cracks in the matrix. By limiting the maximum $J_{tip}$ of the matrix and selecting an appropriate $J_{b}^{\prime }$, guidance can be obtained for the fiber contents of the matrix, interface, and material [37]. The multiple cracking behavior and ductility saturation of the cementitious composites matrix are positively correlated with the PSH index [41]. If the PSHE ($J_{b}^{\prime }/J_{tip}$) and PSHs ($\sigma_{0}/\sigma_{c}$) indices are slightly greater than 1, multiple cracks can appear in the matrix. Most of them are in the unsaturated state, and the tensile strain capacity of the matrix decreases. Therefore, the matrix of these two indicators should have a surplus, otherwise the matrix cannot produce multiple cracking behavior. Kanda and Li [42] considered that $\sigma_{0}/\sigma_{c}>1.3$ and $J_{b}^{\prime }/J_{tip}>2.7$ can ensure saturated PSH behavior in cementitious composites. If the conditions of the PSHE and PSHs cannot be satisfied simultaneously, the matrix exhibits a local fracture. The PSHE and PSHs values are useful for the design of cementitious composites.

This model accurately reflects the strain hardening performance of cementitious composites and provides guidance for practical production applications. The results of the model are close to the actual values, but the interaction integral must be used to introduce the auxiliary field, and the calculation process is complex. At present, there is no effective index for controlling the degree of fracture saturation at the design stage; thus, further research is necessary.

The –(OH) groups in PVA fibers can form a chemical bond ($G_{d}$) with the matrix, affecting the $J_{b}^{\prime }$ of the matrix. $J_{b}^{\prime }$ decreases linearly with an increase in $G_{d}$ [43]. $G_{d}$ also affects then σ–δ curve of the matrix. The crack bridging force of the PVA fiber in the matrix is mainly used to construct a model of the pullout process for a single fiber in the matrix. The pullout process of the fiber is mainly divided into the bonding and pullout stages [44,45]. In this model, it is generally assumed that the fibers are randomly distributed in the matrix [46].

**Figure 8:**
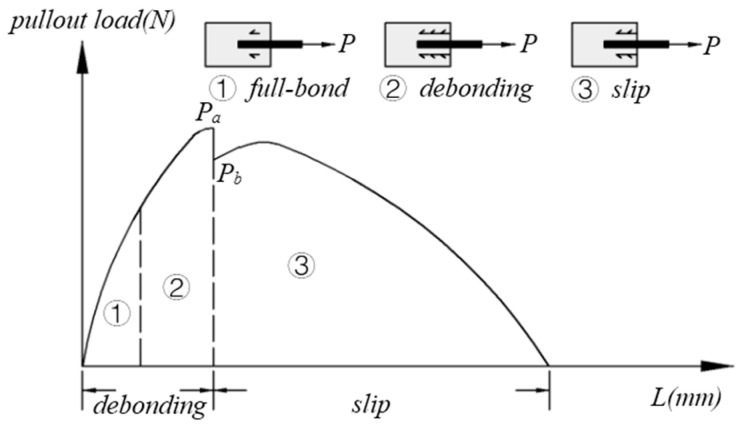
Image of a single fiber being pulled out.

As shown in Figure 8, when the cementitious composite is subjected to an external load of $\leq P_{a}$ at the beginning, the PVA fiber in the base is in the bonding stage. When the load decreases from $P_{a}$ to $P_{b}$, the chemical bond between the PVA fiber and the matrix is broken, and the de-bonding criterion of the fiber in the matrix conforms to the fracture criterion. When the external load is $\geq P_{b}$, the PVA fiber exhibits the phenomenon of slip-hardening in the matrix. The fiber is pulled out when the load exceeds the tensile strength of the fiber [45].

$G_{d}$, the friction stress ($\tau_{0}$) and the slip-hardening coefficient (β) are the three parameters of this model. There is no $G_{d}$ in the matrix of SF or polypropylene fibers, only $\tau_{0}$ and $P_{a}\approx P_{b}$ and β≤0 for matrices containing SFs, polypropylene fibers, and PE fibers. $G_{d}$ decreases with reductions in the $Al^{+3}$ and $Ca^{+2}$ concentrations in the matrix. This is because the –(OH) in PVA fibers can form ionic bonds with $Al^{+3}$ and $Ca^{+2}$ in the matrix. $\tau_{0}$ can be used to simulate the fracture phenomenon caused by the matrix interface slippage. $\tau_{0}$ is mainly affected by the compactness, stiffness, and roughness of the interfacial transition zone between the fiber and the matrix. A larger $\tau_{0}$ indicates that the fiber and the matrix can better resist the slippage of the pulled fiber, but increasing $\tau_{0}$ increases the breakage probability of the fiber during the pullout process and reduces $J_{b}^{\prime }$ [47]. The interfacial micromechanical parameters $G_{d}$ and τ are defined by Equations (17) and (18), respectively:

(17) $$G_{d}=\frac{2(P_{a}-P_{b})}{\pi^{2}E_{f}d_{f}^{3}}$$

(18) $$\tau =\frac{P_{b}}{\pi d_{f}l_{e}}$$

where $E_{f}$ represents the fiber modulus, $d_{f}$ represents the fiber diameter, and $l_{e}$ represents the embedded length of the fiber in the matrix. The drawing process of a single fiber can accurately reflect the function of the fiber in the matrix. The single-fiber pullout model provides a basis for improving the ductility of fiber cementitious composites. However, it does not consider the effect of the fiber content on the matrix. Further research on different hybrid fiber pullout processes is necessary.

### 2.5. DKF Model

Xu and Reinhardt reported that the propagation of the virtual crack at the stress-free crack end extends the nonlinear part of the load–crack mouth opening displacement (P-CMOD) curve. Both the stress-free crack length and the virtual crack length of the equivalent elastic propagation are effective crack lengths. This is the linear progressive superposition hypothesis. Its main purpose is to make the theory of LEFM applicable to the extended description of concrete fracture. It reflects the nonlinear characteristic of concrete cracks and can be used to determine the DKF parameter [48].

**Figure 9:**
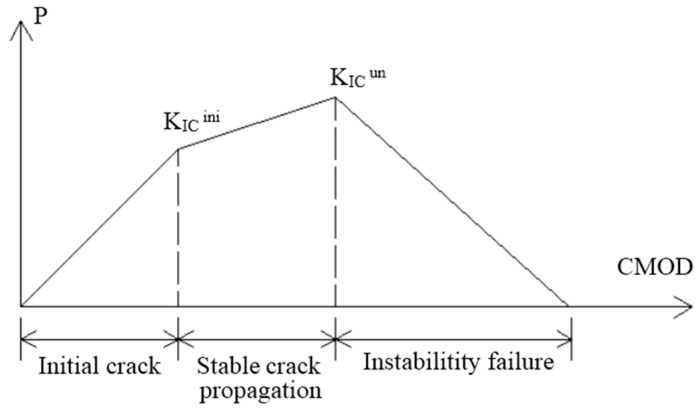
P-CMOD curve of concrete.

On the basis of linear superposition and the FC model, Xu and Reinhardt equated the crack length at any moment after concrete crack initiation to the sum of the elastic stress crack length and the elastic virtual crack length, establishing the DKF model. They considered the effect of the cohesive force in the fracture process area. This model is mainly aimed at solving the characterization problem for the entire process of concrete fracture [49]. The DKF model introduces the initial fracture toughness $K_{Ic}^{ini}$ and the unstable fracture toughness $K_{Ic}^{un}$, which can describe two different fracture transients of concrete. $K_{Ic}^{ini}$ (the control parameter) is calculated using LEFM, according to the initial load ($P_{ini}$) of the crack and the initial crack length $\alpha_{0}$. The fracture toughness calculated using the peak load $P_{Max}$ and the corresponding critical CMOD via the same LEFM equation is called $K_{Ic}^{un}$. The DKF model divides the concrete fracture process into three stages: crack initiation, stable expansion, and unstable expansion, as shown in Figure 9, it can be expressed as follows [48]:
$K_{I}<K_{Ic}^{ini}$Elastic Stage; No Crack$K_{I}=K_{Ic}^{ini}$Crack initiation$K_{Ic}^{ini}<K_{I}<K_{Ic}^{un}$Crack stable propagation$K_{I}=K_{Ic}^{un}$Crack begins to destabilize and expand$K_{I}>K_{Ic}^{un}$Crack instability propagation

**Figure 10:**
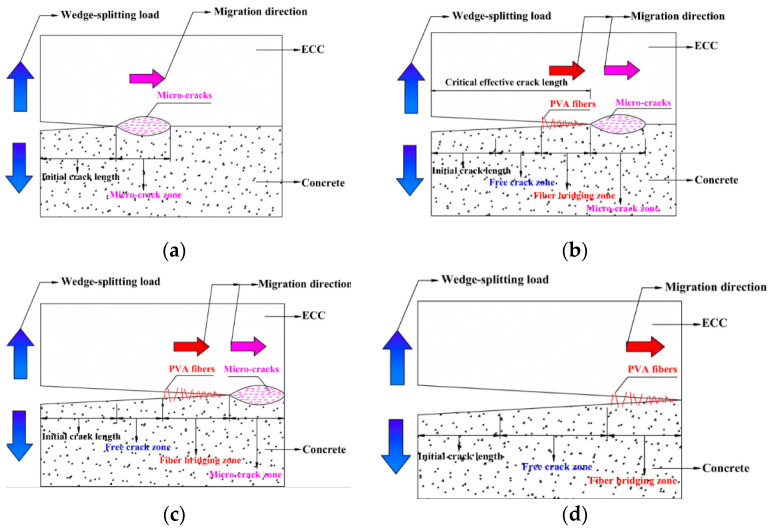
Fracture analysis with four phase (non-linear) [50]. (**a**) Initial crack, (**b**) stable crack propagation, (**c**) instability, and (**d**) instability.

The matrix itself has micro-cracks, and when the matrix is subjected to the external splitting load to a certain extent, the micro-cracks will eventually form a large crack, and P-CMOD also starts to show nonlinearity (Figure 10a). The fiber bridging zone and the micro-crack zone at the crack tip in the matrix also lead to the nonlinear fracture of the matrix (Figure 10b). While in the micro-crack area, large cracks eventually formed until the end of the specimen (Figure 10c), and only fiber bridged cracks were found in the final matrix (Figure 10d). Therefore, the fiber bridged zone and the micro-crack zone at the crack tip in the matrix are the characteristics of its nonlinear fracture [50]. Therefore, the fracture process of the double-K model can be used to easily judge the state of the matrix.

In practical engineering applications, $K_{I}<K_{Ic}^{ini}$ ($K_{I}$ represents the stress intensity factor of a type I fracture) can be regarded as the basis for fracture expansion evaluation of important structures. $K_{Ic}^{ini}<K_{I}<K_{Ic}^{un}$ can be considered as a symptom that occurs before the cracks in important structures are unstable. $K_{I}=K_{Ic}^{un}$ can be used as the evaluation standard for the crack propagation in common structures. Both $K_{Ic}^{ini}$ and $K_{Ic}^{un}$ are correlated with the fracture toughness $K_{I}^{c}$ of the cohesive force in the matrix. Researchers have measured $K_{Ic}^{ini}$ and $K_{Ic}^{un}$ via three-point bending tests [49] and wedge splitting tests [51], and there are also DKF model fracture parameters determined only by the peak load without obvious error [52,53]. Therefore, knowing the values of $K_{Ic}^{ini}$, $K_{Ic}^{un}$ and $K_{I}$ makes it easy to determine the fracture stage of the concrete.

The advantage of the wedge test is that the weights in the calculation process have minimal effects on the fracture energy. This test is suitable for the DKF model. In other methods, e.g., three-point bending, the weight affects 40–60% of the fracture energy [30]. Kumar and Barai [54,55] used the weight function to determine the double-K fracture parameters, which can simplify the calculation of the DKF parameters and avoid special numerical integration. Analyses and comparisons of different methods have been performed, and it was found that different methods can yield similar values for the DKF parameters. A size effect analysis revealed that the depth of the specimen had little effect on its unstable fracture toughness and crack initiation toughness [51].

$K_{Ic}^{ini}$ and $K_{Ic}^{un}$ decrease with the increasing temperature (0–600 °C) [56]. When the loading rate of the specimen ranges from $10^{-5}$ to $10^{-2}$ m/s, with an increase in the loading rate, $K_{Ic}^{ini}$ increases linearly, and $K_{Ic}^{un}$ first increases and then becomes stable [57]. Low sustained loading increases $K_{Ic}^{ini}$ but does not affect $K_{Ic}^{un}$ [58]. When the water pressure is ≤0.3 MPa, $K_{Ic}^{ini}$ is hardly affected by the water pressure, whereas $K_{Ic}^{un}$ increases with an increase in the water pressure [59]. Therefore, the DKF parameters are not affected by the test method but are affected by external environmental factors such as the temperature, water pressure, and loading rate.

The DFK model involves two fracture parameters and a certain size condition, and there is no size effect. The fracture parameters can be determined via a simple test, which can effectively reflect the fracture performance of concrete. The DFK model does not require complex numerical calculations, is simple and easy to understand, and has good application value in practical engineering. However, the DFK model is not suitable for linear fracture propagation. It cannot estimate the critical point of crack initiation to stable propagation; thus, further research in this area is necessary.

The FC, CB, and DFK models are macroscopic nonlinear fracture mechanics models of concrete. The DFK model combines the advantages of the FC and CB models and overcomes their disadvantages to some extent. The FC and DKF models predicted nearly the same fracture behavior for dimensionally notched three-point curved beams, even though they adopted completely opposite principles with regard to the stress singularity at the crack tip [60]. The ACK and PSH models are used mainly for the ductile material of the cementitious composite. The PSH calculation formula is independent of the integral path. These models play an important role in analyzing the stability of cracks in cementitious composite structures, determining the harmfulness of cracks, judging the necessity and effectiveness of engineering reinforcement, and improving the analysis and design methods for ensuring the stability of engineering structures. In this study, the ACK, FCM, PSH, CB, and DFK models were compared, as shown in Table 1.

## 3. Effect of PVA Fiber on Fracture Properties

According to fracture mechanics theory, the fracture energy and fracture toughness of cementitious composites are important indices for measuring the fracture performance of materials. They can reflect the difficulty of crack propagation in matrix materials. Concrete is a brittle material; adding fiber to concrete can improve the fracture property of the matrix. The fiber has a bridging effect on the micro-cracks and macro-cracks in the matrix, controlling the cracking of the matrix and improving the fracture toughness of the matrix [61]. The degree of the bridging action of the fiber is mainly determined by the binding action between the fiber and the matrix and the mechanical bite force [62]. The bridging function of the fiber is also related to the type of fiber and the amount of fibers. Therefore, different fiber types and amounts have different effects on the fracture performance of cementitious composites. The fibers mixed in concrete generally include PVA fiber [63], SF [62], nylon fiber [64], and so on. More commonly, PVA fiber and SF are added to cementitious composites. This is because PVA fiber has the characteristics of high elastic modulus, high strength, acid and alkali resistance, and lower cost than other fibers, which makes PVA fiber widely used in cementitious composites materials. The strength and elastic modulus of SF are generally higher than other fibers, which can also improve the mechanical properties of the matrix. Therefore, this paper mainly analyzes the influence of PVA fiber and SF on the fracture performance of cementitious composite.

### 3.1. Effect of Fiber Content on Fracture Properties

**Figure 11:**
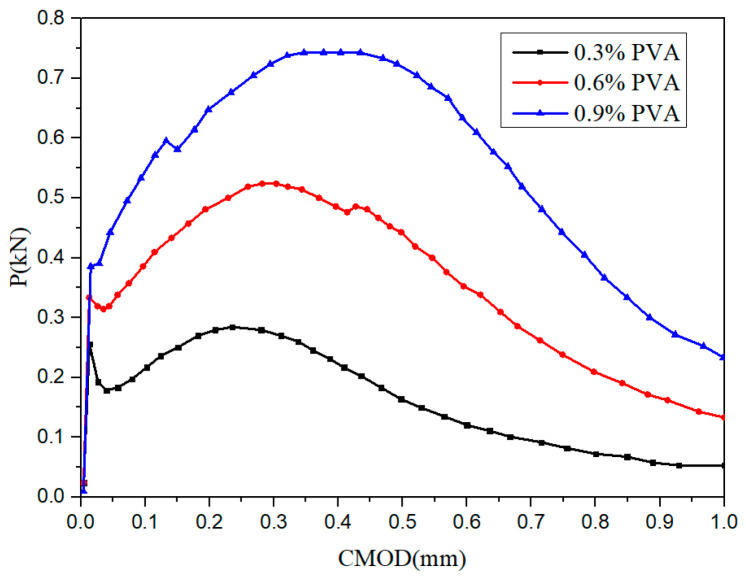
Effect of the PVA fiber content on the P-COMD curve [65].

Toutanji et al. reported that when the PVA fiber content increased from 0% to 0.9%, the fracture performance of cementitious composites gradually increased [63] in agreement with the results of Li et al. (Figure 11) [65]. In the process of reinforcing the matrix, the first crack in the matrix leads to strain softening or local softening. Additionally, the bridging action of the PVA fiber leads to an increase in the hardening stage and limits the crack propagation. When the external load is equal to the fiber strength, the fiber is pulled out or broken, the main crack in the matrix is opened, and the load is reduced (softening stage), resulting in the formation of multiple cracks. However, in one study, the matrix did not exhibit multiple cracks, but only one main crack, possibly because the PVA fiber content was too low (0.3%) to provide a good bridging effect [66].

With a PVA fiber content of 0.6–0.9%, the fiber bridging effect is good, which can be useful for crack control [65]. In four-point bending tests, Zhang et al. found that when the PVA content increased from 0% to 1.2%, the fracture energy, initial fracture toughness and unstable fracture toughness of cementitious composites increased by 1372%, 152%, and 59.18%, respectively. As the PVA fiber content continued to increase to 1.5%, the fracture properties of the matrix were gradually degraded [67]. Adding an appropriate amount of PVA fibers to the matrix can improve its fracture toughness and fracture energy, possibly because PVA fibers can bear external loads during the process of being pulled, play a bridging role, alleviate the stress concentration in the matrix, and limit the generation of new cracks [68]. An excessive amount of PVA fibers in the matrix causes an uneven distribution of the fibers in the matrix, resulting in fiber agglomeration and a large amount of space in the matrix, which degrades the fracture performance of the matrix [69].

Therefore, adding an appropriate amount of PVA fibers (1.5%) to the matrix can improve its fracture performance, but an excessive amount of PVA fibers degrades the fracture performance of the matrix. In engineering applications, the optimal amount of PVA fibers should be determined according to the actual situation, which can reduce the project cost.

### 3.2. Effect of Fiber Length on Fracture Properties

**Figure 12:**
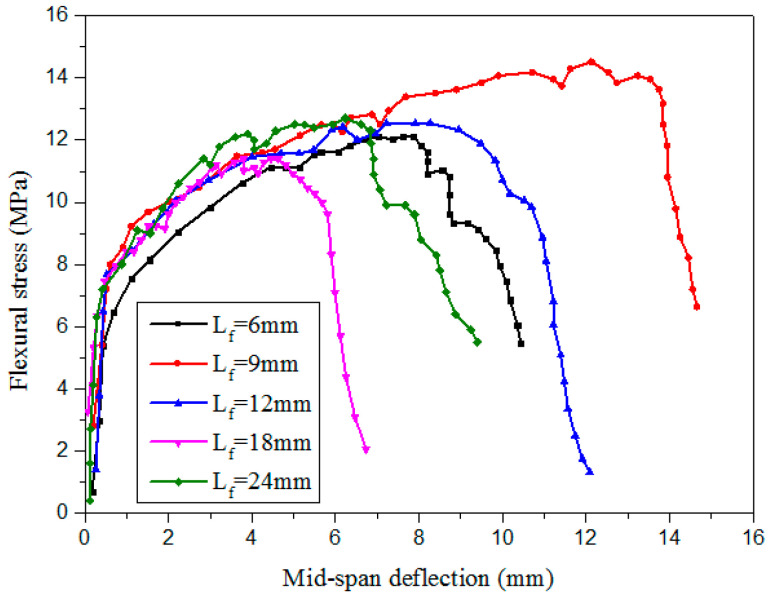
Effect of the PVA fiber length on the flexural strength vs. mid-span deflection curve of the matrix [72].

With an increase in the fiber length, the flexural strength of cementitious composites increased, but the fracture energy of the matrix first increased and then decreased. The fiber length corresponding to the peak fracture energy of the cementitious composite is the optimal fiber length [70,71]. Ding et al. [72] conducted a four-point bending test and a uniaxial tensile test with PVA fiber lengths of 6, 9, 12, 18, and 24 mm. The PVA fiber content in the cementitious composites was 2%. The ultimate flexural strength, ultimate mid-span deflection, and fiber energy dissipation ($G_{f}$) were examined. The results indicated that as the fiber length increased from 6 to 9 mm, the ultimate flexural strength, ultimate mid-span deflection, and $G_{f}$ gradually increased, and with a further increase in the fiber length, the ultimate flexural strength, ultimate mid-span deflection, and $G_{f}$ decreased. When the PVA fiber was 9 mm, $G_{f}$ was maximized (144.2 kJ/m^3^), and the ultimate flexural strength was 13.9 MPa (Figure 12).

**Figure 13:**
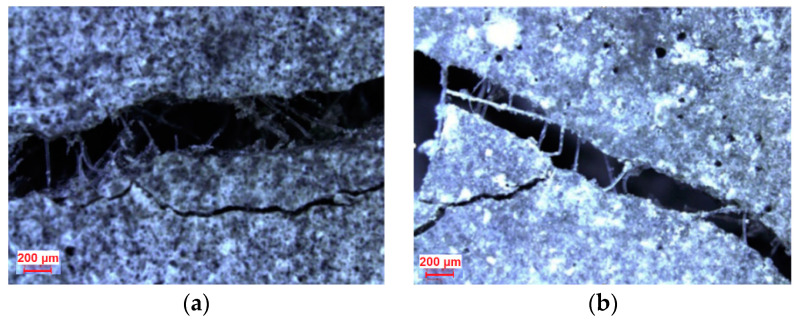

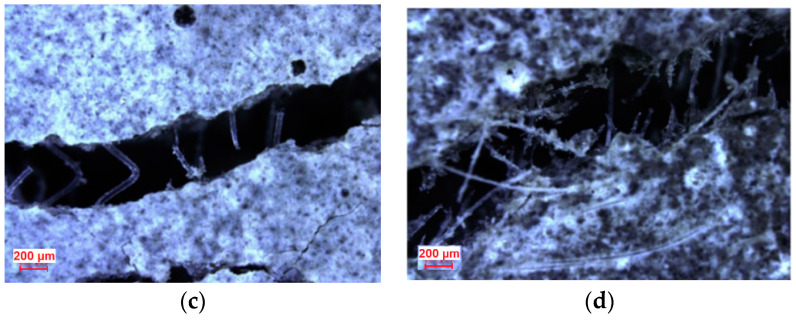
Microscopic images of cracked beams with different fiber lengths and volume fractions, including bridging fibers [73]. (**a**) PVA length of 8 mm and fiber content of 1%, (**b**) PVA length of 12 mm and fiber content of 1%, (**c**) PVA length of 8 mm and fiber content of 2%, and (**d**) PVA length of 12 mm and fiber content of 2%.

Sasmal and Avinash [73] studied the effects of the PVA fiber length 8 and 12 mm on the fracture properties of the matrix. They found that compared with 12 mm PVA fibers, 8 mm fibers resulted in a higher fracture energy of and a smaller number of fractures after bridging. After the addition of 1% PVA fibers to the matrix, the number of fiber fractures was small (Figure 13). It is considered that with the proper length and mixing amount, the fibers were more evenly distributed in the matrix and absorbed a larger amount of fracture energy, but the fibers did not transfer energy. It is also believed that the fracture toughness of PVA fibers with a length of 16 mm is good [74]. If the fibers are too short, the bridging effect on the cracks is not strong. The addition of fibers that are too long and easily dispersed unevenly in the matrix can lead to matrix fracture [70]. PVA fibers with different lengths have different effects on the fracture properties of the matrix. Generally, the optimal PVA fiber length is 16 mm. Further research should be performed to determine the optimal PVA fiber length according to the maximum fracture energy of the of the *σ*-*δ* curve.

### 3.3. Effect of Fiber Surface Oiling on Bond Performance

The hydrocarbonyl group in PVA fibers is highly hydrophilic and can form strong chemical bonds with the cementitious composites. Thus, the PVA fibers in the matrix are prone to fracture when the matrix is subjected to an external load, limiting the phenomena of multiple cracking and strain hardening in cementitious composites [45]. Therefore, surface treatment of PVA fiber is required to reduce the chemical bonding between the fiber and the surrounding cementitious composites. Studies have revealed that oiling the surface of PVA fibers can weaken the chemical bonds between the PVA fibers and the matrix and significantly improve the ductility of PVA-cementitious composites [43].

**Figure 14:**
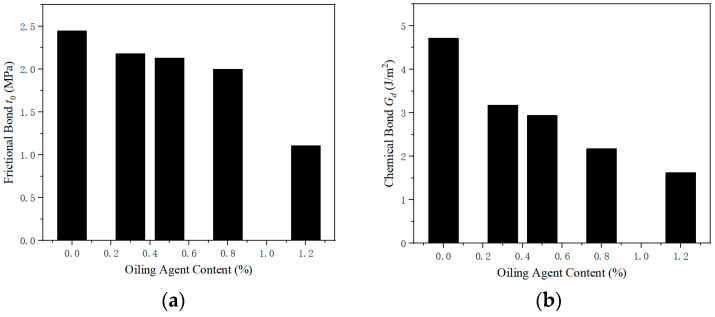
Effect of the fiber oil content on the bond performance of the matrix [43]. (**a**) Frictional bond, (**b**) chemical bond.

**Figure 15:**
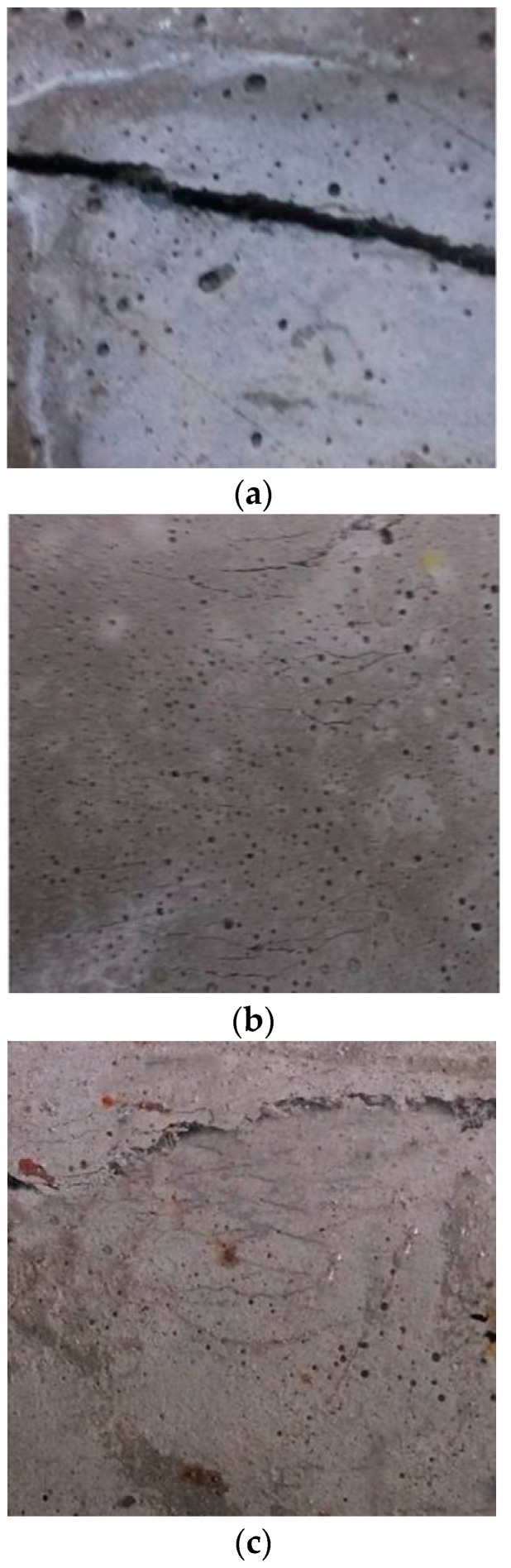
Failure mode of matrix [76]. (**a**) Mortar, (**b**) oil-coated PVA, and (**c**) PVA coated without oil.

With an increase in the amount of oil applied to the surface of the PVA fibers, both $\tau_{0}$ and $G_{d}$ decreased (Figure 14) [43]. The quality dosage of the PVA fiber surface mastering oil was 1.2% of fiber quality,$\tau_{0}$, and $G_{d}$ decreased by about approximately 30%. This is because oiling can weaken the bonds between the PVA fibers and the matrix, making the fibers easy to pull out [45]. With 1.2% PVA in the matrix, the $J_{b}^{\prime }$ and $\sigma_{0}$ increased by 34.2 J/m and decreased by 0.5 MPa, respectively, compared with those without oil, and the tensile ductility of PVA-cementitious composites oiled on the surface was improved [75]. Under the same load, the elastic modulus of the oil-coated PVA fiber is large; thus, the deformation of the cementitious composite matrix is small. The cracks in the matrix of oil-containing PVA-cementitious composites are smaller than those for composites without oil, but the width of the cracks is ≤50 μm (Figure 15) [76]. Both $\tau_{0}$ and $G_{d}$ decreased with an increase in the amount of oil applied to the PVA fiber surface. The best quality of the coating oil was 1.2% of the PVA fiber quality. If the surface of PVA fiber is oiled, the optimal PVA fiber content can be 2%.

## 4. Effect of SFs on Fracture Properties

The flexural strength, splitting strength, durability, toughness, and energy absorption of cementitious composites can be improved by adding an appropriate amount of SFs to the matrix [77,78,79]. When cementitious composites are subjected to loads and cracks, the SFs randomly distributed in the matrix intersect with the crack surfaces, bridging the cracks [80]. During the de-bonding and pullout of SFs, the pulling force hinders the crack propagation, increasing the energy consumption and improving the toughness of the matrix [81]. In the fracture process of SF cementitious composites, the friction between the SFs and the matrix is overcome. Because the tensile strength of the SFs is significantly higher than the friction, the SFs are generally pulled out. 

According to Lee et al., the content of arch-type SF in the matrix is the same content as that of hooked-end SF, and the fracture energy of cementitious composite mixed with arch-type SF is 1.34–2.98 times that of hooked-end SF [82]. The fracture energy of an arch-type SF was higher than that of a hooked-end SF with the same volume, indicating that the cementitious composites of arch-type SFs have higher fracture resistance energies. This may be because the hooked-end SF can be pulled out more easily than the arch-type SF, resulting in faster reduction of the stress in the cracked matrix [83].

**Figure 16:**
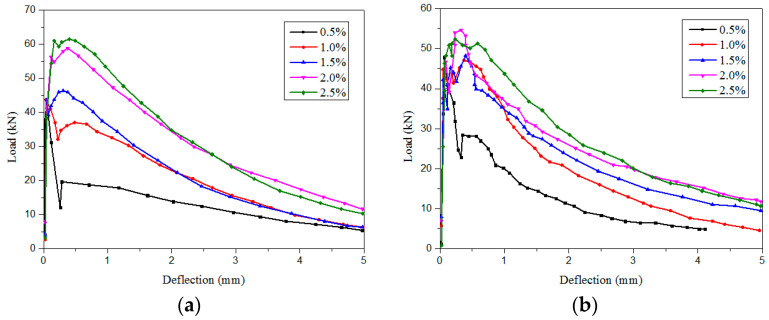
Effects of the SFs content and type on the load–deflection curve [84]. (**a**) Micro-straight SFs (%), (**b**) hooked-end SFs (%).

**Figure 17:**
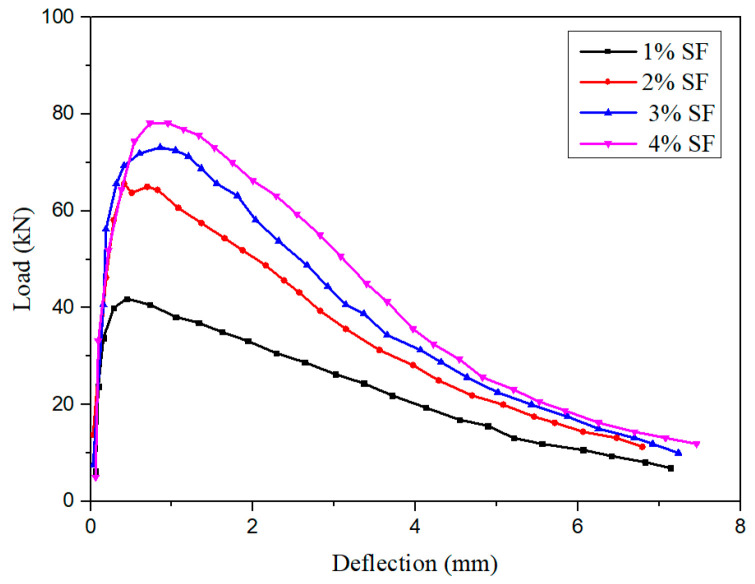
Effects of the SFs content on the load–deflection curve [86].

Ren et al. [84] performed four- and three-point bending tests to examine the effects of micro-straight and hooked-end SFs on the fracture performance of cementitious composites within the dosage range of 0–2.5%. The results indicated that the type and dosage of SFs hardly affected the initial cracking strength and deflection of the cementitious composites, but significantly influenced the load–deflection curve after cracking (Figure 16). The maximum load increased with the SF content, possibly because the fibers increased the bearing capacity of the matrix. This is similar to the results of Wu et al. and Yoo et al. [85,86] (Figure 17). The fracture toughness of the matrix (the critical stress strength of the material during fracture failure) increased with an increase in the SF content. Micro-straight SFs have a greater effect on the breaking energy than hooked-end SFs. The optimal SF content depends on the substrate.

**Figure 18:**
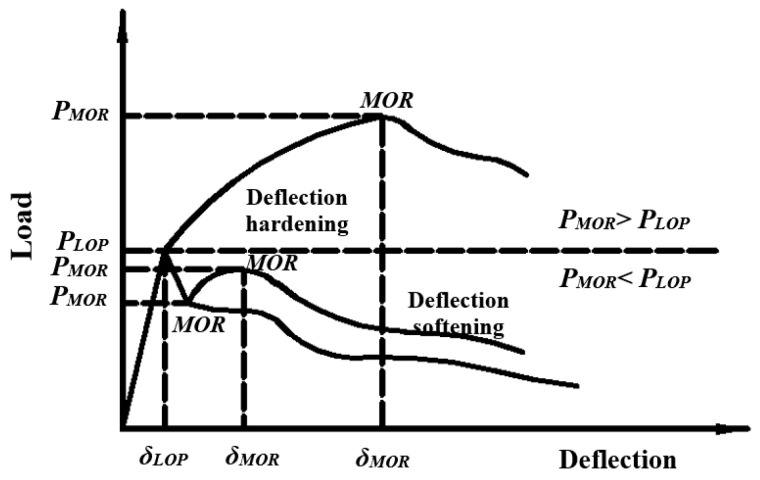
Typical load–deflection curves [87].

The four-point flexural load-deflection curves are typical. In Figure 18, “MOR” represents the modulus of rupture, and “LOP” represents the limit of proportionality. According to the matrix, for the first time, the changes in the bearing capacity of the cracking and the bending capacity of the matrix are divided into deflection hardening and softening stages. The first cracking point is an obvious nonlinear point in the load–deflection curve. The equivalent energy given by the area under the load–deflection curve is defined as the initial cracking toughness, and the bearing capacity (equal bending strength), energy absorption capacity (toughness), and cracking behavior (crack number) of the matrix can be determined [87]. Because they are easy to understand, the typical load–deflection curves are widely used.

Won et al. [88] considered that micro-straight SFs have a better enhancement effect on the flexural strength and toughness of the matrix than hooked-end SF, because there are more micro-straight SFs than hooked-end SFs per unit volume added, and micro-straight SFs have better anti-cracking performance. The load–displacement curve decreases rapidly after cracking, possibly because the micro-straight SFs are smaller than the hooked-end SFs and have a lower strength under shear action. Additionally, the micro-straight SFs have poor bonding performance with the matrix; thus, they are easily pulled out, whereas the hooked SFs are easily broken.

**Figure 19:**
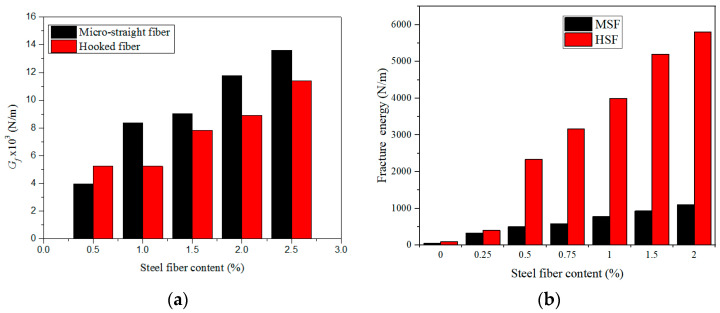
Effects of the SF fiber type and content on $G_{f}$ [84,89]. (**a**) Ratio of water to cementitious materials: 0.16, (**b**) water–binder ratio: 0.195.

With an increase in the SF content (from 0 to 2.5%), the fracture energy of the matrix gradually increased (Figure 19). As shown in Figure 19a, the micro-straight SF length was 13 mm, and the hooked-end SF length was 25 mm, with an SF content of >1%. Adding 2.5% micro-straight SFs caused a significantly larger increase in the fracture energy of the matrix than adding 2.5% hooked-end SFs. The maximum fracture energy of the cementitious composites with 2.5% micro-straight SFs was approximately 1.2 and 3.5 times higher, respectively, than those for 2.5% hooked-end SFs and 0.5% micro-straight SFs. This is because the number of micro-straight SFs per unit volume was large, and the fiber length was sufficient for bridging the cracks and absorbing energy [84].

As shown in Figure 19b, the micro-straight SF length was 6 mm, and the hooked-SF length was 30 mm. The micro-straight SF length was sufficiently short, and the hooked SF length was sufficient for connecting macro-cracks, which increased the amount of energy needed for specimen destruction. However, micro-straight SFs cannot bridge large cracks, or provide a stable post-peak response. Hooked SFs can bridge large cracks, which improves the ductility of cementitious composites, the bending strength related to the fracture energy, and the stability of the post-peak response [89,90]. In the range of 0–4% SFs, the fracture energy of the matrix increased gradually with the increasing fiber content. The fracture property of cement matrix composites can be greatly improved by adding 4% SF. However, the effect of the fiber type is different for different matrices. Adding SFs to the matrix can improve the fracture energy of the matrix, because SFs can enhance the crack resistance and absorb energy.

## 5. Effects of Hybrid Fibers on Fracture Performance

Traditional cementitious composites employ only PVA fibers. To improve the mechanical properties and durability of cementitious composites, researchers developed mixed fiber cementitious composites; generally, more than two types of fibers are mixed [91]. Hybrid fibers can be mixed with different types, lengths, elastic moduli, and tensile strengths. Long fibers can bridge large cracks, and short fibers can bridge micro-cracks. The bridging action of low-modulus fibers prevents the propagation of large cracks, which results in multiple cracks [92]. The performance of the cementitious composites is mainly affected by the fiber characteristics and amount, the matrix performance, and the interaction between the fibers and the matrix [91].

Adding an appropriate amount of high-modulus and low-modulus fibers to cementitious composites can make the cracks of the matrix smaller, increase the stress–strain capacity, and enhance the bending toughness, tensile strength, and friction strength compared with cementitious composites with one kind of fiber [93,94,95]. This is known as the hybrid fiber effect; i.e., the performance of hybrid fibers mixed with cementitious composites is significantly better than that of a single type of fiber mixed [96,97]. Owing to the large difference in elastic modulus between SF and PVA fibers, proper mixing of the two in cementitious composites can improve the mechanical properties of the matrix and produce a positive hybrid effect.

Zhang et al. [98] studied the effects of the SF dosage and water–binder ratio on the bending resistance of PVA-cementitious composites. The results indicated that with an increase in the water-cement ratio (from 0.25 to 0.55), the critical deflection of 1.7% PVA-cementitious composites increased from 1.0 to 4.71 mm. With an increase in the SF content, the cracking strength and bending strength of PVA-ECC increased, and the enhancement became more significant with a decrease in the water–binder ratio. Additionally, the SF has an obvious bridging effect on the second peak load in the fracture model of the matrix, and the first peak load is only affected by the fracture toughness or strength of the matrix. Therefore, the synergistic effects of hybrid fibers can improve the fracture performance of cementitious composites.

**Figure 20:**
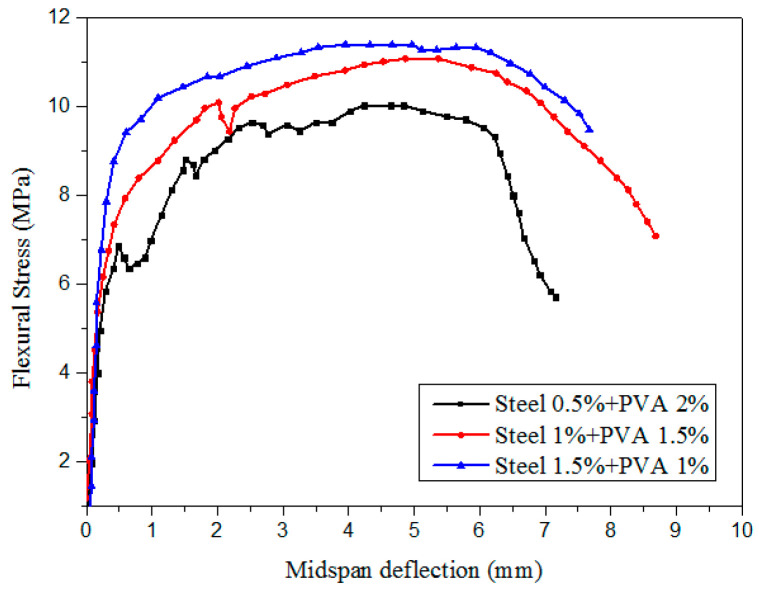
Effects of SFs and PVA fibers on the flexural strength and mid-span deflection of cementitious composites [99].

**Figure 21:**
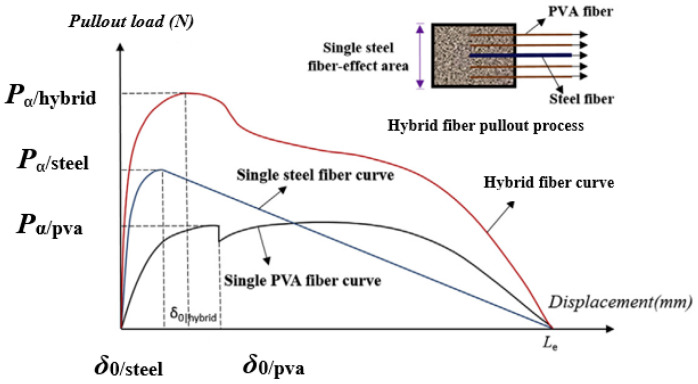
Load–displacement curve for the pullout of a single fiber [100].

As shown in Figure 20, the initial crack strength and ultimate strength of SF and PVA fiber-reinforced cementitious composites increased with the SF content. SFs are more effective than PVA fibers for improving the initial cracking strength and ultimate strength of the matrix, possibly because the stiffness of SFs is higher than that of PVA fibers. SFs with high stiffness can improve the bending strength of the matrix, and PVA fibers with low stiffness can improve the deflection of the matrix [93,99]. When cementitious composites are subjected to an external load, the pullout load for SFs Pa|steel is larger than that for PVA fibers Pa|PVA. Moreover, SFs have a good ability to prevent the development of cracks. The synergistic effects of SF and PVA fibers on the matrix are shown in Figure 21 [100].

The fracture property of the matrix can be improved by appropriately adding SFs and PVA fibers to the matrix, owing to their synergistic effects. The toughness index (I) refers to the area under the load–deflection curve prior to a given deflection, divided by the area under the same curve prior to the first cracking [101]. Hybrid fiber-cementitious composites with I_5_ > 5, I_10_ > 10, and I_30_ > 30 are also strain hardening composites. The load–displacement curve for the pullout of a single fiber can be used to analyze the synergistic effects of mixed fibers on the matrix, as well as the synergistic effects of other fibers. Generally, adding 1.5% SFs and 1.0% PVA fiber to the matrix can greatly improve the fracture performance of the matrix. The total dosage of PVA fiber and SF should ≤ 3%. This satisfies the modification of SFs and PVA fibers to cementitious composites.

## 6. Conclusions

The fracture models of cementitious composites and the effects of PVA fibers and SFs on the fracture properties of cementitious composites were examined. According to a correlation analysis, the following conclusions are drawn.

(1) The fracture model of the cementitious composite, the ACK model, and the PSH model are used mainly for ductile cementitious composites. ACK model can determine the optimal fiber content of matrix, PVA fiber generally no more than $J_{b}^{\prime }/J_{tip}>1$ is the basic condition for the quasi-strain hardening of cementitious composites, while a large value of $J_{b}^{\prime }/J_{tip}$ can realize the cracking of multi-saturated cracks in the matrix. The FC, BC, and DKF models are mainly applied to semi-brittle material–concrete composites. Both FC and BC models are based on tensile softening of concrete, and the value of $G_{f}$ can be expressed as the ability of concrete to resist crack propagation. The FC model can directly simulate the concrete with nonlinear characteristics by using the finite element method. The DKF model has the advantages of the FC and BC models, and is widely used in practical engineering. The DKF model can judge the whole process of concrete failure according to $K_{Ic}^{ini}$, $K_{Ic}^{un}$, and $K_{I}$ parameters.
(2) The fracture energy and toughness of the cementitious composite can be increased by adding an appropriate amount of PVA fibers (1.2–2.0%) with an appropriate (16 mm) fiber length. The main objectives of PVA fiber surface oiling (approximately 1.2%) are to weaken the adhesion between the PVA fibers and the matrix, reduce the friction during fiber pullout, and prevent the fracture of PVA fibers.
(3) The stiffness of SFs is higher than that of PVA fibers; thus, SFs have a better strengthening effect on the cementitious composite. SF fibers of appropriate length have a good bridging effect on macroscopic cracks in the matrix, and SFs can also absorb fracture energy. The mixing amount of SF in is cementitious composite 4%, which can greatly improve the fracture property of the matrix. In the pullout process, the SF is easily pulled out, whereas the PVA fiber is easily broken. SF and PVA fibers can be mixed in the matrix simultaneously. The pullout load displacement of the fibers is the synergistic effect of the two. Generally, adding 1.5% SFs and 1.0% PVA fiber to the matrix can greatly improve the fracture performance of the matrix. Thus, adding appropriate amounts of PVA fibers and SFs can improve the mechanical and fracture properties of the matrix.

## 7. Outlook

(1) For comparison with the ACK model, the PSH, FC, BC, and DKF models are reviewed and analyzed. Although all the models have advantages, there are deficiencies for practical engineering that must be resolved.
(2) However, when PVA fiber and SFs fiber are mixed, the optimal effect of SF and PVA fiber on the fracture property, mechanical property, and durability of the matrix is not discussed in depth. In the study of SFs and PVA fiber mixing, too much range of water–binder ratio was not set, and too much research on its high-temperature performance was not carried out. The pullout of a single fiber’s load–displacement curves of polypropylene fibers and SF single fibers were also not analyzed. To improve the mechanical properties of cementitious composites with low fiber contents, further research should be performed in these areas.
(3) The fracture properties of fiber-reinforced cementitious composites are studied and analyzed only with regard to the basic properties and mechanisms. Therefore, it is necessary to further study the practical application of cementing composites and the freezing–thawing environment in which the cementing materials are located, as well as the wet, hot, and salt environment in saline and alkaline areas and coastal areas.

## Figures and Tables

**Table 1:** Comparison of the five models for cementitious composites.

Model	Crack Criterion	Mechanical Treatment of Micro-Fissure Zone	Numerical Method Used
ACK model [22]	$V_{f}\geq V_{c}$ $\sigma_{f}^{\prime }>\sigma_{m}^{u}$	Stress–strain curve	Integral method
FC model [27]	$\sigma \geq f_{t}$ $w=w_{0}$	Bilinear (or nonlinear) strain softening curve	Finite-element method
CB model [34]	$\sigma \geq f_{t}$ $w=w_{0}$	Micro-cracks are uniformly distributed and parallel and the damage degree is expressed by the reduction of the elastic modulus	Finite-element method
PSH model [39]	$J_{b}^{\prime }>J_{tip}$	Energy under quasi-stress strain	Integral method
DFK model [52]	$K_{I}=K_{Ic}^{ini}$	Load–CMOD curve	Weight functions and other numerical calculation methods

Note: $V_{c}$ represents the critical volume of the fiber.
